# Knee Diameter and Cross-Section Area Measurements in MRI as New Promising Methods of Chondromalacia Diagnosis-Pilot Study

**DOI:** 10.3390/medicina58091142

**Published:** 2022-08-23

**Authors:** Dominik Sieroń, Izabella Jabłońska, Dawid Lukoszek, Karol Szyluk, Hugo Meusburger, Georgios Delimpasis, Maciej Kostrzewa, Ivan Platzek, Andreas Christe

**Affiliations:** 1 Department of Radiology SLS, Inselspital, Bern University Hospital, University of Bern, Freiburgstrasse 10, 3010 Bern, Switzerland; 2 Recreation and Treatment Center “Glinik” 1, Wysowa-Zdrój 101 Street, 38-316 Wysowa-Zdroj, Poland; 3 Dawid Lukoszek Physiotherapy Osteopathy, 42-690 Hanusek, Poland; 4 Department of Physiotherapy, Faculty of Health Sciences in Katowice, Medical University of Silesia in Katowice, 40-752 Katowice, Poland; 5 Department of Orthopaedic and Trauma Surgery, District Hospital of Orthopaedics and Trauma Surgery, Bytomska 62 Street, 41-940 Piekary Slaskie, Poland; 6 Institute of Sport Sciences, The Jerzy Kukuczka Academy of Physical Education, 40-065 Katowice, Poland; 7 Department of Radiology, Dresden University Hospital, Fetscherstrasse. 74, 01307 Dresden, Germany

**Keywords:** chondromalacia, Outerbridge, knee surface area, knee diameter, BMI

## Abstract

*Background and Objectives*: Chondromalacia often affects the knee joint. Risk factors for the development of cartilage degenerative changes include overweight, female sex and age. The use of radiological parameters to assess the knee joint is rarely reported in the literature. *Materials and Methods*: The study involved 324 patients, including 159 (49%) women and 165 (51%) men, with an age range between 8–87 years (mean: 45.1 ± 20.9). The studied group had a body mass index (BMI) in the range of 14.3–47.3 (mean: 27.7 ± 5.02). A 1.5 Tesla and 3.0 Tesla (T) MRI scanner was used to assess the cartilage of the knee joint using the Outerbridge scale. The radiological parameters analyzed were the Insall–Salvati index, knee surface area, knee AP (antero-posterior) maximal diameter and knee SD (sinistro-dexter) maximal diameter. *Results*: Parameters such as the knee surface area, knee AP maximal diameter and knee SD maximal diameter showed a significant correlation with Outerbridge Scale (*p* < 0.014). The age of the patients showed a significant correlation with each knee parameter (*p* < 0.004). Results of knee AP and SD maximal diameter measurements strongly depended on BMI level. *Conclusions*: A significant relationship was found between the knee surface area, knee AP maximal diameter and knee SD maximal diameter and the advancement of chondromalacic changes in the knee joint, age and BMI.

## 1. Introduction

Chondromalacia describes the painful softening and consecutive destruction of the hyaline cartilage that often affects the knee joint [1]. Cartilage responds to environmental changes and mechanical loading [1,2]. In clinical practice, magnetic resonance imaging (MRI) and the modified Outerbridge classification are often used to assess the severity of chondromalacia and the subsequent osteoarthritis [3,4]. The risk factors for knee chondromalacia include overweight, female sex, age, congenital structural defects and acute joint trauma [5]. Chondromalacia is a condition of articular cartilage that affects both athletes and inactive people. In addition, anterior knee pain is common among athletes due to overload or overuse [6].

Imaging measurements are popular in endoprosthesis studies due to the requirement of collecting anthropometric data of the tibia for surgery [7]. The use of measurements such as the Insall–Salvati index in assessing the knee joint for chondromalacia changes and injury risk factors such as anterior cruciate ligament (ACL) injury has been reported in the literature [8,9,10,11]. MR imaging is not instantly available everywhere, and it is also an expensive examination for assessing degenerative knee changes. Therefore, we hypothesized if other measurements such as diameter or surface area of the knee could be a surrogate for Outerbridge classification, since overweight correlates well with Outerbridge classification and should also lead to larger knee diameters.

The purpose of this study was to examine the relationship between knee joint parameters (the Insall–Salvati index, knee surface area, knee AP maximal diameter (antero-posterior) and knee SD (sinistro-dexter) maximal diameter) and chondromalacia changes and to compare these parameters to other known risk factors for knee joint degeneration such as age, sex and body mass index (BMI). An additional objective was to note differences in the Outerbridge classification between 1.5 Tesla and 3.0 Tesla (T) MRI (a higher resolution).

## 2. Materials and Methods

### 2.1. Ethics

The institutional review board (IRB) approval could be waived due to the retrospective nature of the study, including irreversible anonymization of patient identifiers.

### 2.2. Study Design

In the current observational cross-sectional study, we analyzed the association between knee joint measurements (Insall–Salvati, knee surface area, knee SD (sinistro-dexter) maximal diameter, knee AP (antero-posterior) maximal diameter) and chondromalacia cartilage lesions, including demographic variables (age, sex) and BMI, in a continuous group of patients undergoing knee MRI in 2018 and 2019. Patients were recruited from community and clinical hospitals and private institutions in Zamość Elblag, Jelenia Góra and Bielsko-Biala (Poland). The study was performed according to STROBE guidelines. Evaluation of cartilage chondromalacia included the medial (femur medial, tibia medial), lateral (femur lateral, tibia lateral) and anterior (femur, patella) compartments of the knee joint.

### 2.3. Inclusion and Exclusion Criteria

Any patients with knee pain who underwent a MRI of the knee at the indicated radiological institutes were consecutively included in this study. The analyzed group of patients was referred by orthopedists, surgeons or rehabilitation specialists owing to complaints of pain or suspicion of arthrosis or post-traumatic lesions. Regarding pain complaints, individuals reported their own request for examination in the scope of private services. The study group consisted of 324 patients: 159 (49.1%) women and 165 (50.9%) men. A total of 155 (47.8%) patients, including 70 (45.2%) women and 85 (54.8%) men, were examined using the 1.5 T unit; 169 (52.2%) patients, including 89 (52.7%) women and 80 (47.3%) men, were examined using the 3.0 T scanner. Four age classes were defined for the study: <30 years—94 participants; 30–45 years—61 participants; 46–60 years—78 participants; >60 years—91 participants.

Exclusion criteria: patients with previous surgery or chronic post-traumatic changes were excluded from this study.

### 2.4. Evaluation of Cartilage Chondromalacia

To evaluate cartilage chondromalacia, we employed the 4-level Outerbridge classification (Table 1) using fat-saturated proton density sequences - a modified classification for arthroscopic cartilage evaluation [12,13,14,15].

### 2.5. Knee Parameter Measurements

The following parameters of the knee joint were evaluated in the study:

**Insall–Salvati index**—an index describing the height of the patella in the knee joint based on the ratio of the length of the patellar ligament to the length of the patella in the cranio-caudal dimension.

**Figure 1:**
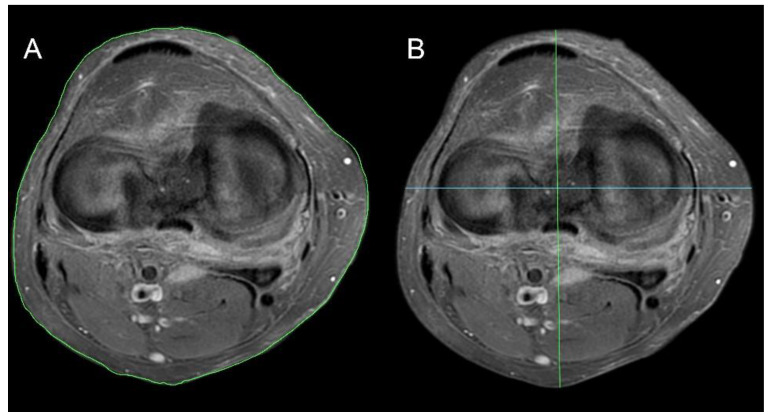
Knee joint parameter methodology. (**A**) Knee surface area, (**B**) knee AP maximal diameter (green line) and knee SD maximal diameter (blue line).

**Knee surface area**—the surface of the knee (cross-sectional area of the leg) measured at the level of the femo-tibial joint space. The calculation algorithm of the knee surface is based on the OSIRIX MD program (Pixmeo SARL, 266 Rue de Bernex, CH-1233 Bernex, Switzerland, Figure 1A).

**Knee maximal SD diameter**—the maximum dimension of the knee at the level of the femoral-tibial joint in the left to right dimension (S-sinistro, D-dexter) (Figure 1B, green line).

**Knee maximal AP diameter**—the maximum dimension of the knee at the level of the femoral-tibial joint space in the left to right dimension (sinistro-dexter) (Figure 1B, blue line).

### 2.6. Image Acquisition

MRI was performed using a 3.0 T scanner (Ingenia 3.0T, Philips, Amsterdam, Netherlands) or a 1.5 T GE scanner (SIGNA, GE, Milwaukee, WI, USA) at different facilities located in clinical hospitals and private facilities in Zamość Elbląg, Jelenia Góra and Bielsko-Biala.

The following diagnostic sequence protocol was used in the study: axial, sagittal and coronal PD FS; sagittal and coronal T1 (all with a slice thickness of 3 mm); and 3D high-resolution PD FS with a slice thickness from 0.8 to 1 mm.

Data were evaluated using iMac pro (Apple, Cupertino, CA, USA) with FDA-approved OsiriX MD software (version 11.0, Pixmeo SARL, Bernex, Switzerland). All MRI analyses were irreversibly anonymized.

### 2.7. Statistical Analysis

Data were statistically analyzed for differences between sex (χ^2^ test), and 1.5 T and 3.0 T apparatus types (χ^2^ test). A χ^2^ test was used to check for significant differences between age subgroups, Outerbridge scale scores and knee joint parameters (Insall–Salvati, knee surface area, knee SD maximal diameter and knee AP maximal diameter). Spearman rank correlation was assessed. The relationship between BMI level and the results of knee joint parameters (Insall–Salvati, knee surface area, knee SD maximal diameter and knee AP maximal diameter) was evaluated using R Spearman rank correlation (rho). A correlation coefficient (rho) < 0.4 was considered as a weak correlation and a rho > 0.6 meant a strong correlation; in between these values, the correlation was defined as moderate [17]. The above analysis was performed for the whole group, for the 1.5 T and 3.0 T apparatus, and for women and men separately. The significance level was set at *p* < 0.05.

## 3. Results

### 3.1. Demographics

The 159 women and the 165 men were on average 48.6 ± 23.1 and 43.0 ± 20.4 years old and had an average BMI of 27.4 ± 5.6 and 27.9 ± 4.3. 

### 3.2. Outerbridge Scale

Table 2 shows the mean and median measurements of the knee in the entire study population.

An increase in the knee surface area or an increase in any maximal diameter (antero-posterior and sinistro-dexter) is associated with an increased Outerbridge classification (*p* < 0.0014, Table 3). There was no significant correlation between the Insall–Salvati index measurement and the Outerbridge score for each knee joint compartment (*p* > 0.5). The statistical analysis showed a significant positive correlation between the other three knee measurement Outerbridge scores for each knee joint compartment (Table 3). The knee surface area demonstrated, on average, the highest correlation with the Outerbridge score, followed by the knee SD maximal diameter and knee AP maximal diameter. The best correlation with the Outerbridge Scale was found in the medial compartment, followed by the anterior compartment and then the lateral compartment.

### 3.3. Age

**Figure 2:**
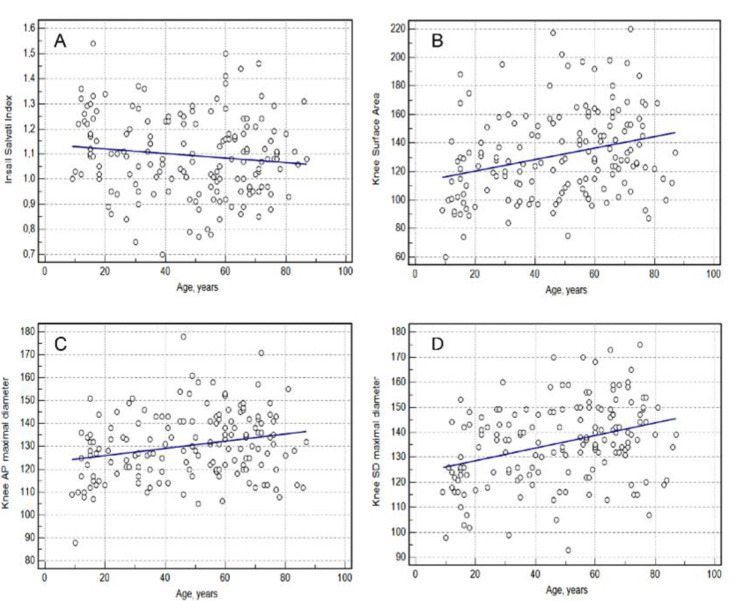
Relationship between knee measurement and age. (**A**)—Insall Salvati index; (**B**)—Knee Surface Area; (**C**)—Knee AP (anterior-posterior) Maximal Diameter, (**D**)—Knee SD (sinistro-dexter) Maximal Diameter.

Age demonstrated a significant correlation with all knee measurements (Spearman rank correlation, *p* < 0.05). The Insall–Salvati index, knee surface area, knee AP maximal diameter and knee SD maximal diameter showed correlation coefficients of rho = −0.1447, rho = 0.2056, rho = 0.1597 and rho = 0.2287, respectively (Table 4) (Figure 2).

### 3.4. Sex

There was no statistically significant difference in the Insall–Salvati index scores (*p* = 0.4869), knee surface area (*p* = 0.1046) or knee SD maximal diameter (*p* = 0.5356) between the female and male subjects, obtained using both apparatuses.

**Figure 3:**
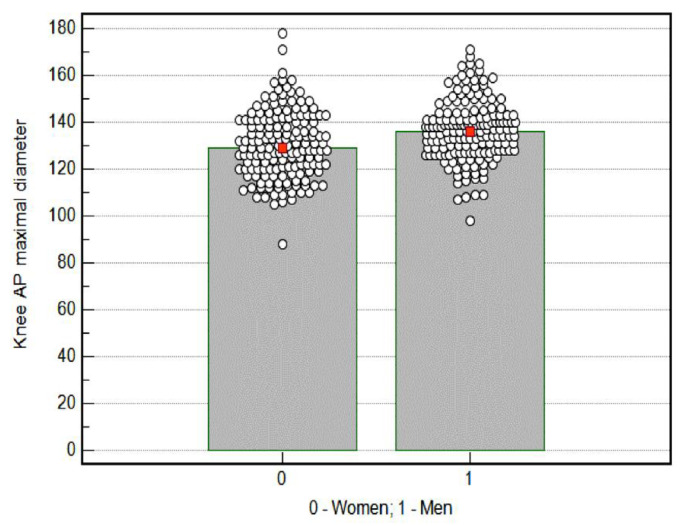
Knee AP (anterior-posterior) maximal diameter results by sex. Red mark—mean; cicular mark—participants.

The average value of knee AP maximal diameter obtained using both devices among women was statistically significantly lower (130.4 mm) than the value obtained among men (136.5 mm), *p* = 0.0001 (Figure 3).

### 3.5. BMI

The average BMI for the entire study group was 27.7 ± 5.02. Table 5 shows the average BMIs for Outerbridge scores of 0, 1, 2, 3 and 4 in the examined articular surfaces of the knee joint.

There was no mutual correlation between BMI and the Insall–Salvati index (*p* = 0.3980).

**Figure 4:**
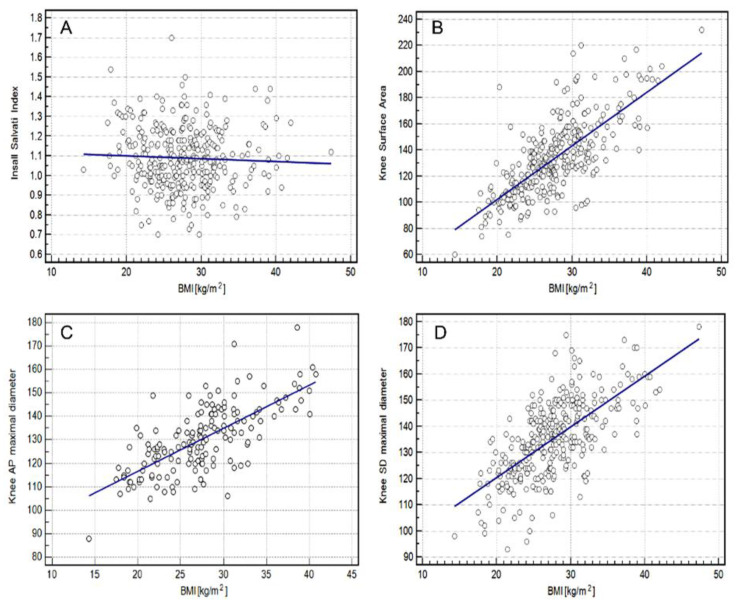
Relationship between BMI (Body Mass Index) and knee measurement using both scanners (1.5 T and 3.0 T) in the study group. (**A**)—Insall Salvati index; (**B**)—Knee Surface Area; (**C**)—Knee AP (anterior-posterior) Maximal Diameter, (**D**)—Knee SD (sinistro-dexter) Maximal Diameter.

The highest correlation between BMI and knee measurements was found for the knee surface area (rho = 0.7023, *p* = 0.0001), followed by the knee SD maximal diameter (rho = 0.6498, *p* = 0.0001) and the knee AP maximal diameter (rho = 0.6375, *p* = 0.0001) for the whole group using both scanners (Figure 4).

### 3.6. Comparison of MRI 1.5 T with MRI 3.0 T

The maximal Outerbridge score per patient at the 1.5 T MRI was 2.38 ± 1.46 and not significantly different from the 3.0 T MRI (2.29 ± 1.58, *p* = 0.71). The average value of the Insall–Salvati index parameter obtained using the 1.5 T apparatus was statistically significantly higher than that obtained using the 3.0 T apparatus (*p* = 0.0001). A similar result was obtained for the knee SD maximal diameter (*p* = 0.0038). The average value of the knee surface area (*p* = 0.3925) and knee AP maximal diameter (*p* = 0.7257) obtained using 1.5 T was comparable to that obtained using 3.0 T.

## 4. Discussion

In the present study, we observed a weak association between the knee surface area, knee AP maximal diameter and knee SD maximal diameter parameters and the Outerbridge scale in each knee joint compartment. Knee joint measurements were significantly associated with age. The Insall–Salvati index decreased with increasing age. In contrast, other parameters showed larger measurements in older subjects. The only difference between men and women was found in the knee AP maximal diameter, with women having lower scores. A strong, significant relationship was found between BMI and the proposed knee joint parameters, with the exception of the Insall–Salvati index.

The measurement of the diameters of the knee is a quick tool to assess the degeneration of the knee joint, not only on MR images but also on conventional X-ray images, ultrasound and computed tomography. Furthermore, clinicians can directly measure the diameters or the circumference of the knee on the patient without imaging. Cut-off values of these measurements for a certain degree of degeneration are part of ongoing studies.

The Insall–Salvati index provides an assessment of patella alignment, which can reflect pain in the anterior compartment of the knee joint [18]. The literature reports results that point to patella alta as the cause of diseases such as Osgood–Schlatter, patellofemoral joint instability and chondromalacia. A study by Kar et al. showed that patella infera (0.86) was associated with chondromalacia of the patella [19]. In the current study, there was no significant relationship between the Outerbridge score (chondromalacia classification) and the Insall–Salvati index (1.1 ± 0.15). Özel, on the other hand, noted that both variants of patella positioning can lead to chondrolysis, and low patella positioning has been overlooked when considering predisposing factors due to its rare occurrence [20]. Additionally, the results of this study indicate a trend for the Insall–Salvati index measurement to decrease with age, which also fits with the progression of chondromalacia lesions. It seems that by using the Insall–Salvati index, a group of conditions detected by the analysis of MRI findings can be quickly covered [18,19,21,22].

Clinical examination of the knee joint for osteoarthritis begins with observation of an enlarged joint contour [23]. We found that the study of knee joint contour widening in knee osteoarthritis lesions is not common. Some literature reports thickening of the knee joint circumference due to swelling or edema after hip or knee endoprosthesis, which may cause arthrogenic limitation of knee joint extension and flexion [24,25]. Pain, morning stiffness, limitation of function and thickening of the joint contour are considered to be the most important symptoms in the diagnosis of osteoarthritis of the knee [26]. In this study, the knee surface area, knee AP maximal diameter and knee SD maximal diameter were much more associated with BMI than with the Outerbridge scale. The most likely explanation for these differences is the change in knee joint geometry with age and the progression of degenerative changes in the joint.

Due to the frequent occurrence of cartilage disease in athletes, individualized approaches to managing knee dysfunction and careful observation are recommended [27]. In the case of inactive people, quadriceps strengthening is recommended as one of the approaches to prevent chondromalacia and subsequent osteoarthritis of the knee [28]. It should be emphasized that the current study may be of significant clinical importance in the diagnosis of knee dysfunction, especially in people practicing sports, both recreationally and professionally.

### 4.1. Strengths

The large amount of 324 patients with six cartilage regions examined in the knee joint lead to robust statistical results. The current study may be of significant clinical importance in the diagnosis of knee dysfunction, especially in people practicing sports, both recreationally and professionally.

### 4.2. Limitations

One of the major limitations of the study is the lack of an asymptomatic control group. In addition, information such as thickening of the knee joint bursa, Hoffa’s pad and fluid in the joint cavity, which can significantly affect radiographic findings, were not included in the analysis. The population examined at the 1.5 T and 3.0 T was pooled together for all the non-MRI unit-related analysis, which may have confounded the results, since the 3.0 T unit is supposed to have the higher resolution and with that a possible different Outerbridge classification. The lack of previous studies on radiographic parameters of the knee joint in terms of BMI, chondromalacia severity, age and sex limited the reference to the repeatability of the measurements and results.

## 5. Conclusions

The radiological parameters used in this study, such as the knee surface area, and knee AP and SD maximal diameter can be used to confirm chondromalacia on MR imaging.

These measurement parameters can help in the clinical assessment of chondromalacia of the knee, not only by measuring the knee on the images but also by directly measuring the knees of patients. 

## Figures and Tables

**Table 1:** Outerbridge classification [16].

Grade	Macroscopy	MRI
Grade 0	Normal cartilage	Normal cartilage
Grade 1	Rough surface; chondral softening, focal thickening	Inhomogeneous; high signal; surface intact; cartilage swelling
Grade 2	Irregular surface defects; <50% of cartilage thickness	Superficial ulceration, fissuring, fibrillation; <50% of cartilage thickness
Grade 3	Loss of >50% cartilage thickness	Ulceration fissuring, fibrillation; >50% of depth of cartilage
Grade 4	Cartilage loss	Full thickness chondral wear with exposure of subchondral bone

**Table 2:** Measurement characteristics of the group in terms of knee joint measurement results (SD: standard deviation, Q: quartile).

Parameter	Mean	SD	Median	Q25	Q75
Insall–Salvati Index	1.1	0.15	1.08	0.99	1.18
Knee Surface Area	133.8	28.27	131.00	114.00	149.00
Knee AP Maximal Diameter	133.5	13.90	134.00	125.00	142.00
Knee SD Maximal Diameter	135.4	14.61	135.00	124.00	145.50

**Legend:** SD, standard deviation; Q25, lower quartile; Q75, upper quartile.

**Table 3:** Relationship between knee measurement and Outerbridge Scale in each compartment of the knee.

		Knee Surface			Knee AP Maximal Diameter			Knee SD Maximal Diameter		
Surface	N	Spearman	t(N-2)	*p*	Spearman	t(N-2)	*p*	Spearman	t(N-2)	*p*
Femur Lateral	324	0.2234	4.1120	0.0000	0.1950	3.5673	0.0004	0.2021	3.7025	0.0003
Femur Medial	324	0.3067	5.7823	0.0000	0.2647	4.9259	0.0000	0.2615	4.8625	0.0000
Tibia Lateral	324	0.2098	3.8503	0.0001	0.1771	3.2289	0.0014	0.1928	3.5259	0.0005
Tibia Medial	324	0.2975	5.5925	0.0000	0.2655	4.9411	0.0000	0.2636	4.9030	0.0000
Patella	324	0.2351	4.3394	0.0000	0.1962	3.5900	0.0004	0.2160	3.9695	0.0001
Femur	324	0.2472	4.5780	0.0000	0.2071	3.7981	0.0002	0.2074	3.8052	0.0002

**Legend:** N, number of observations; *p*, statistical significance; t(N-2), Recurrence Relation.

**Table 4:** Relationship between knee measurement and age.

Correlating Variables	N	Spearman	T(N-2)	*p*
Insall–Salvati Index and Age	324	−0.1447	−2.6243	**0.0002**
Knee Surface Area and Age	324	0.2056	3.7691	**0.0000**
Knee AP Maximal Diameter and Age	324	0.1597	2.9022	**0.0040**
Knee SD Maximal Diameter and Age	324	0.2287	4.2152	**0.0000**

**Legend:** N, number of observations; p, statistical significance; t(N-2), Recurrence Relation.

**Table 5:** BMI for the Outerbridge scale.

Outerbridge	BMI (Mean ± SD)					
	*Femur Lateral*	*Tibia Lateral*	*Femur Medial*	*Tibia Medial*	*Femur*	*Patella*
Grade 0	26.0 ± 4.88	25.7 ± 4.92	25.3 ± 4.47	25.4 ± 4.55	25.3 ± 4.30	25.0 ± 3.81
Grade 1	29.1 ± 5.31	28.8 ± 4.97	26.4 ± 4.17	28.2 ± 4.71	29.0 ± 5.90	26.4 ± 5.05
Grade 2	29.4 ± 4.49	29.6 ± 4.54	29.4 ± 3.85	28.3 ± 3.97	28.8 ± 4.09	29.2 ± 5.29
Grade 3	29.6 ± 5.00	28.8 ± 4.06	29.9 ± 4.90	28.3 ± 3.97	30.6 ± 4.09	29.5 ± 4.53
Grade 4	27.0 ± 3.54	29.4 ± 5.33	30.3 ± 5.01	29.6 ± 4.78	29.4 ± 5.29	29.8 ± 4.56

**Legend:** BMI, Body Mass Index; SD, standard deviation.

## Data Availability

The data are available upon special request.
